# Three-dimensional mapping in multi-samples with large-scale imaging and multiplexed post staining

**DOI:** 10.1038/s42003-023-04456-3

**Published:** 2023-02-03

**Authors:** Siqi Chen, Guangcai Liu, Anan Li, Zhixiang Liu, Ben Long, Xiaoquan Yang, Hui Gong, Xiangning Li

**Affiliations:** 1 Britton Chance Center for Biomedical Photonics, Wuhan National Laboratory for Optoelectronics, Huazhong University of Science and Technology, Wuhan, 430074 China; 2 Research Unit of Multimodal Cross Scale Neural Signal Detection and Imaging, Chinese Academy of Medical Sciences, HUST-Suzhou Institute for Brainsmatics, JITRI, Suzhou, 215125 China; 3 Key Laboratory of Biomedical Engineering of Hainan Province, School of Biomedical Engineering, Hainan University, Haikou, 570228 China

**Keywords:** Neuroscience, Imaging

## Abstract

Dissection of the anatomical information at the single-cell level is crucial for understanding the organization rule and pathological mechanism of biological tissues. Mapping the whole organ in numerous groups with multiple conditions brings the challenges in imaging and analysis. Here, we describe an approach, named array fluorescent micro-optical sectioning tomography (array-fMOST), to identify the three-dimensional information at single-cell resolution from multi-samples. The pipeline contains array embedding, large-scale imaging, post-imaging staining and data analysis, which could image over 24 mouse brains simultaneously and collect the slices for further analysis. With transgenic mice, we acquired the distribution information of neuropeptide somatostatin neurons during natural aging and compared the changes in the microenvironments by multi-component labeling of serial sections with precise co-registration of serial datasets quantitatively. With viral labeling, we also analyzed the input circuits of the medial prefrontal cortex in the whole brain of Alzheimer’s disease and autism model mice. This pipeline is highly scalable to be applied to anatomical alterations screening and identification. It provides new opportunities for combining multi-sample whole-organ imaging and molecular phenotypes identification analysis together. Such integrated high-dimensional information acquisition method may accelerate our understanding of pathogenesis and progression of disease in situ at multiple levels.

## Abstract (web summary)

A new method, named array fluorescent micro-optical sectioning tomography (array-fMOST), is developed for identifying the three-dimensional information at single-cell resolution from multi-samples.

## Introduction

Dissection the living organism systemically need the morphology and location information of the numerous cells which have unique physiological and pathological phenotype^1^. As for the cells have individual coordinate in extremely complex connections, the acquisition of the whole-organ anatomical information in three dimensions is crucial for comprehensively understanding the cellular organization logic and pathologic mechanism^2–4^. As the most complex organ, the brain contains trillion-level neurons, glia and other types of cells with complex connectivity. It implies that the functional states of different cells vary across distant regions, requiring large-scale investigations^5,6^. The whole-organ mapping efforts are building the foundations for the understanding of tissue architecture at the mesoscopic level^7^. Traditionally, 3D information of biological samples was obtained by reconstructing series of 2D images of mechanical thin sections^8^. The low throughput of this approach makes the data reconstruction and analysis a tedious task.

Several techniques with automated whole-organ imaging methods were developed to overcome this problem^9^. A series of imaging techniques enable fast data acquiring of the whole-tissue, such as the light-sheet combined with the optical clearing technique^10,11^. However, it needs multiple processes for several days to make the tissues transparent before imaging. Another type of whole-organ imaging technique is serial section tomography as fMOST^12–14^ and STP^15,16^, which could image at the high resolution in three dimensions for the neuron reconstruction in the whole brain. However, the individual differences between biological samples make it necessary to analyze multiple samples for comparison with statistical analysis. The present single-cell level imaging methods all need long time for the tissue processing and imaging over one or more weeks for individual whole-brain of mouse.

Moreover, the preservation of anatomical structure and biomarker is a prerequisite for the true anatomy of biological samples and tissue molecular phenotypes, especially at the cellular or subcellular level^17–19^. The chemical processing such as the delipidation during the tissue-clearing was shown to cause damage to the cellular membrane^20^, or lead to the loss of structural characteristic signals^21^, which may limit the reliability of this method in analyzing the real morphological structure of tissue. Given these limitations, the need for a method, which has advantages in large-scale imaging for parallel experiments in multi-samples, is exemplified in systematic analysis with intact tissue morphological structure and molecular phenotype maintenance.

In this study, we developed a high-throughput system for single-cell level localization and identification of the whole-organ, which includes multi-sample embedding, whole-organ imaging and data processing. With a three-dimensional voxel resolution of 0.65 × 0.65 × 3 μm^3^, 32 mouse brain samples were imaged simultaneously in 15 days, while the slices could be collected for further investigation. Using the methods, we analyzed the degenerated changes in the distribution of somatostatin-positive neurons during natural aging, as well as the location and neuron types in the upstream circuits of the medial prefrontal cortex in the whole brain. These results indicated that the system enables hierarchically performing of cellular localization, molecular phenotype identification and cytoarchitecture dissection at three-dimensional scale of multi-samples.

## Results

### Efficient embedding and imaging implementation of multiple mouse brain

**Fig. 1:**
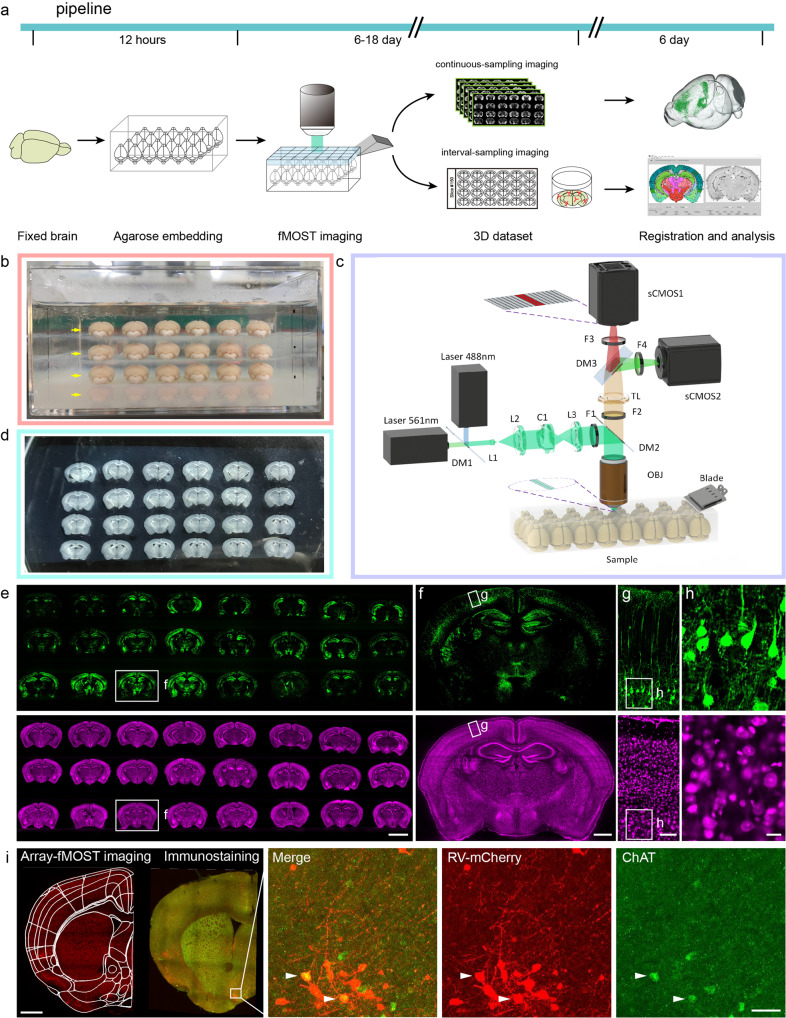
The high-throughput 3D mapping for multiple samples at single-cell resolution. **a** The array-fMOST pipeline, including agarose embedding, fMOST imaging and data analysis. **b** Demonstrating the process of multi-samples embedding. Yellow arrows indicate four-layer array arrangement of multiple samples. **c** The fMOST setup and components of the high-throughput serial sectioning and imaging system. **d** 50 μm brain slices obtained after systematic imaging. **e** Images of whole-brain pyramidal neuron axonal projections and cytoarchitecture from multiple samples acquired through this pipeline (30 µm thickness). **f-h** Magnified view of the boxed region in e is shown. **i** An immunohistochemical analysis of choline acetyltransferase conducted after array-fMOST whole-brain imaging of RV-labelled input neurons of mPFC. Serial-sectioned images at 50 μm intervals and enlarged images are shown on the right. Scale bars: e, 5 mm; f, 1 mm; g, 100 μm; h, 20 μm; i, 1 mm, 50 μm.

In order to achieve high-throughput in situ imaging and analysis of multiple samples under the same conditions, we developed a high-throughput cell localization and identification analysis scheme (array-fMOST) (Fig. 1). The whole process includes multi-sample embedding, high-resolution dual-color imaging, data registration and analysis. Before sample imaging, half a day is required for multi-sample array embedding. The imaging process can be divided into two modes: continuous imaging and interval imaging according to different data acquisition requirements. Continuous imaging was used to obtain continuous three-dimensional data sets of the whole brain, which has unique advantages in the analysis of projection patterns of axon fibers and single neuron reconstruction in the whole brain. The interval imaging mode, not only obtained information on the distribution of whole brain cells, but also realized the collection of complete sections for subsequent protein phenotype identification and multi-component staining. After data registration and analysis, the raw 3D data obtained by this program can be visualized and analyzed quantitatively (Fig. 1a). In the embedding part of multiple samples, we set twenty-four samples in an array arrangement. About three minutes later, the surface of the agarose in the mold begins to solidify, and the agarose around the sample was still in a liquid state. Then, the second group of samples was placed on the surface of the agarose (Fig. 1b). The complete sections of embedded agarose samples could be collected during the imaging process, which can be used for subsequent molecular phenotype identification of various proteins in tissues (Fig. 1d).

The imaging system can realize the automatic acquisition of three-dimensional multicolor information. As shown in Fig. 1c, the imaging system includes two parts: microscope and microtome. The optical microscope part consists of a line-scanning confocal microscope. Image signals excited by two lasers at 488 nm and 561 nm are projected to two sCMOS cameras through a set of optical components for fast acquisition of green and red dual-channel data. The slicing device consists of a self-made high-precision vibrating slicer. During the data acquisition process, where multiple scanning strips can cover the entire surface of the sample, the system acquires the fluorescence images layer by layer. Then, the vibrating microtome slices to remove the imaged layers according to the programmed slice thickness. This cycle of imaging-slicing-imaging is automatically repeating until all data from the sample is acquired. Here, we present images of whole-brain pyramidal neuron axonal projections and cytoarchitecture from multiple samples acquired through this pipeline (Fig. 1e). From the figure, we can clearly see the axon and dendrites of the neuron. Combined with the cytoarchitectural information obtained by PI staining, neuronal cell bodies and brain regions of axonal projections in multiple samples can be accurately located (Fig. 1f–h). After fluorescence imaging with array-fMOST, whole sections can be collected and serial sections can be used for subsequent validation experiments, such as protein molecular phenotyping and super-resolution imaging. Here, we performed an immunohistochemical analysis of choline acetyltransferase conducted after array-fMOST whole-brain imaging of RV-labelled input neurons of mPFC (Fig. 1i).

### Comparison of technologies for whole-brain architectonic dissection

**Fig. 2:**
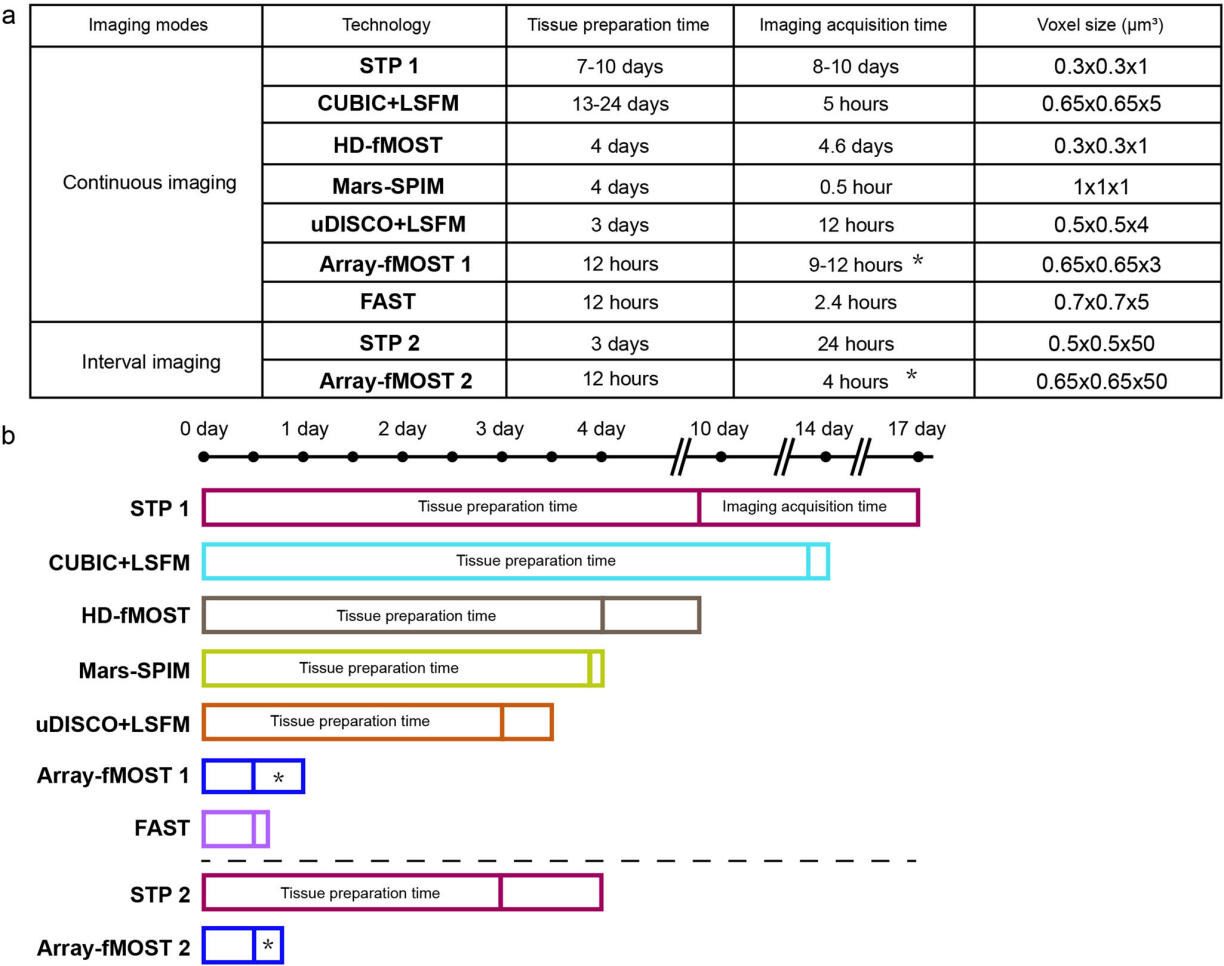
Comparison of technologies for whole-brain architectonic dissection of mice. **a** Detailed data of tissue preparation time, imaging time and voxel size of whole-brain mapping technologies. The tissue preparation time of all methods mentioned here did not include mouse cardiac perfusion, post-tissue fixation and rinsing. Voxel size: for techniques based on 2D scanning of brain sections, the axial voxel size is the spacing between sampled sections. **b** Timeline of different technologies for whole-brain architectonic dissection of a mouse brain. (*: It was stated that the more samples in the agarose, the less imaging time is required for each mouse brain (Supplementary Fig. 1 and Supplementary Table 1). Technologies: STP, serial two-photon tomography; HD-fMOST, high-definition fluorescent micro-optical sectioning tomography; FAST, block-face serial microscopy tomography; CUBIC, clear, unobstructed brain imaging cocktails and computational analysis; uDISCO, ultimate three-dimensional imaging of solvent-cleared organs; LSFM, light-sheet fluorescence microscopy; Mars-SPIM, multiangle-resolved subvoxel selective plane illumination microscope).

Array-fMOST enables fast data acquisition speed and high imaging quality, while achieving the comparison of multiple samples under the same conditions. To compare various tissue processing methods combined with whole-brain imaging technologies^10,12,22–26^, we summarize the time required for each method to acquire a complete 3D dataset of an adult mouse brain at different voxel resolutions, including tissue preparation time and image acquisition time (Fig. 2a). By comparison, we found that the three-dimensional imaging method of the tissue-clearing method using the light-sheet microscope has great advantages in imaging speed. However, the preparation in the early stage of this method often takes a lot of time. In addition, tissue clearing inevitably causes morphological changes in the tissue, hampering accurate geometric measurement. The difference of clearing between the inner and outer part of the brain or among different brains hinder the quantitative analysis with high accuracy. Here, the imaging speed and time consumed were compared based on voxel size. Array-fMOST achieves a balance by reducing tissue processing and cutting time for multiple samples (Fig. 2b). It can complete the acquisition of three-dimensional datasets of thirty-two mouse brains with voxel resolution of 0.65 × 0.65 × 3 μm^3^ in fifteen days. If the resolution was changed to 0.3 μm × 0.3 μm × 1 μm (as for STP1), the imaging time would have been 14 times larger. The original 274 hours required for imaging 24 mouse brains would have increased to 3836 hours, equivalent to 159 hours for one mouse brain. Compared to the 240 hours for one mouse brain in STP1, our method was faster. Moreover, instead of the two-photon laser in STP1, we used LED as the light source, which was cheaper and more applicable in ordinary laboratories. And the more mouse brains were in the agarose, the less imaging time was required on average for a single mouse brain. The above results demonstrate that the system achieves a balance between tissue preparation time, imaging speed, and imaging quality.

### Mapping and comparing various cell-type samples in mice through this pipeline

**Fig. 3:**
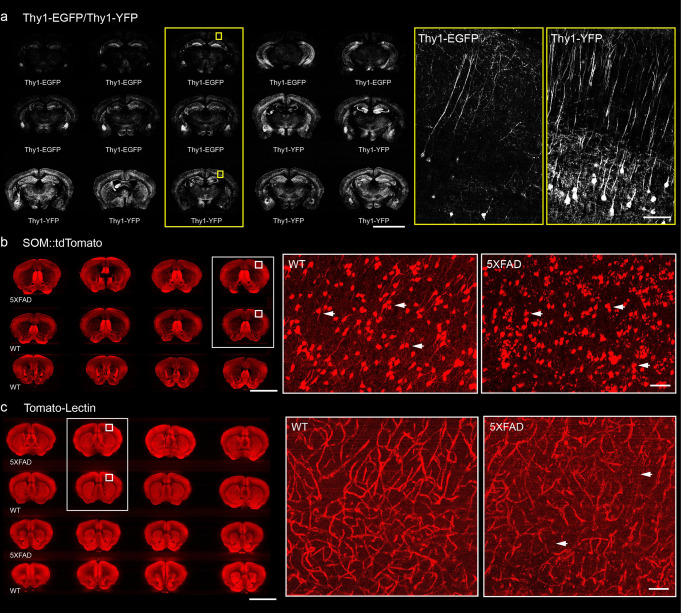
High-throughput and high-resolution whole brain images of various cell-types samples in mice. **a** Comparison the intensity of fluorescent signal between the Thy1-EGFP M-line and Thy1-YFP H-line mice (4 µm thickness). **b** Comparison of SOM-expressing interneurons and long projecting neurons in 5×FAD::SOM::tdTomato mice and SOM::tdTomato mice (100 µm thickness). The white arrows indicate changes on the morphology of neurons. **c** Whole-brain vasculature in mice perfused with fluorescein-labeled tomato lectin between 5×FAD and wild type mice (100 µm thickness). Magnified view of the boxed region is shown on the right. The white arrows indicate the morphology alterations of vessels. Scale bars: a, 5 mm, 100 μm; b, c, 5 mm, 50 μm.

To validate this method in samples with different fluorescent proteins and different labeling strategies, we exhibited contrastive images of multiple samples of different types, including the following: whole-brain projections of Thy1-YFP and Thy1-EGFP mice whose pyramidal neurons express green fluorescent protein with varying degrees of sparseness; cell body distribution and morphological information of SOM-Cre::Ai14 mice expressing red fluorescent signal in somatostatin positive neurons; whole-brain vascular information labeled with Tomato-Lectin (Fig. 3). In the following images, some subcellular structures such as soma and axons of different neurons were clearly visualized. To verify the applicability of this method to samples with different degrees of sparsity, multiple coronal images of Thy1-YFP H-line and Thy1-EGFP M-line mice were obtained after the same tissue processing and imaging parameters (Fig. 3a). By extracting 4 µm thickness data from three mouse brains at once, it can be found that, the long-range projection axons of pyramidal neurons in these brains were clearly visualized. Thy1-YFP H-line mice showed more fluorescent signals and stronger brightness; while the Thy1-EGFP M-line mice were significantly weaker. The range of transmission wavelengths of the green channel filter used in our imaging system is 510 nm to 540 nm. This wavelength range has no significant influence on the imaging of EGFP and YFP proteins. Consistent with previous research^27^, our results showed that there were more fluorescent cells in the Thy1-YFP H-line than in the Thy1-EGFP M-line, which might be caused by the different sparsity of fluorescent protein expression in the brain of different transgenic mice. This indicates that this method can image and compare samples of different fluorescence intensities under the same conditions. Subsequently, array-fMOST performed imaging on multiple samples from SOM-Cre::Ai14 and 5×FAD::SOM-Cre::Ai14 mice, to compare the distribution and number of SOM neurons in different pathological states. SOM-Cre::Ai14 mice which express red fluorescent protein in SOM-positive neurons were used to observe the soma and cellular processes morphology of SOM neurons. As shown in Figs. 3b, 5×FAD::SOM-Cre::Ai14 mice showed abnormal cell morphology in the cortex. Finally, array-fMOST acquired multi-samples vascular information labeled with Tomato-Lectin to validate the imaging on fluorescent dyes, such as Dylight 594. We used 5×FAD mice to characterize Alzheimer’s disease-related disorders. By comparing the vascular structure information of mice with different pathological states, the change of vascular density in the cortex in 5×FAD mice can be clearly visualized (Fig. 3c). These results show that this pipeline can realize the comparison of multiple samples with different types at single-cell resolution under the same conditions, which provides a more efficient way for the analysis and summary of the morphology changes in biological research.

### Whole brain somatostatin neuron mapping and comprehensive immunohistochemical analysis of its microenvironment in aging mice

**Fig. 4:**
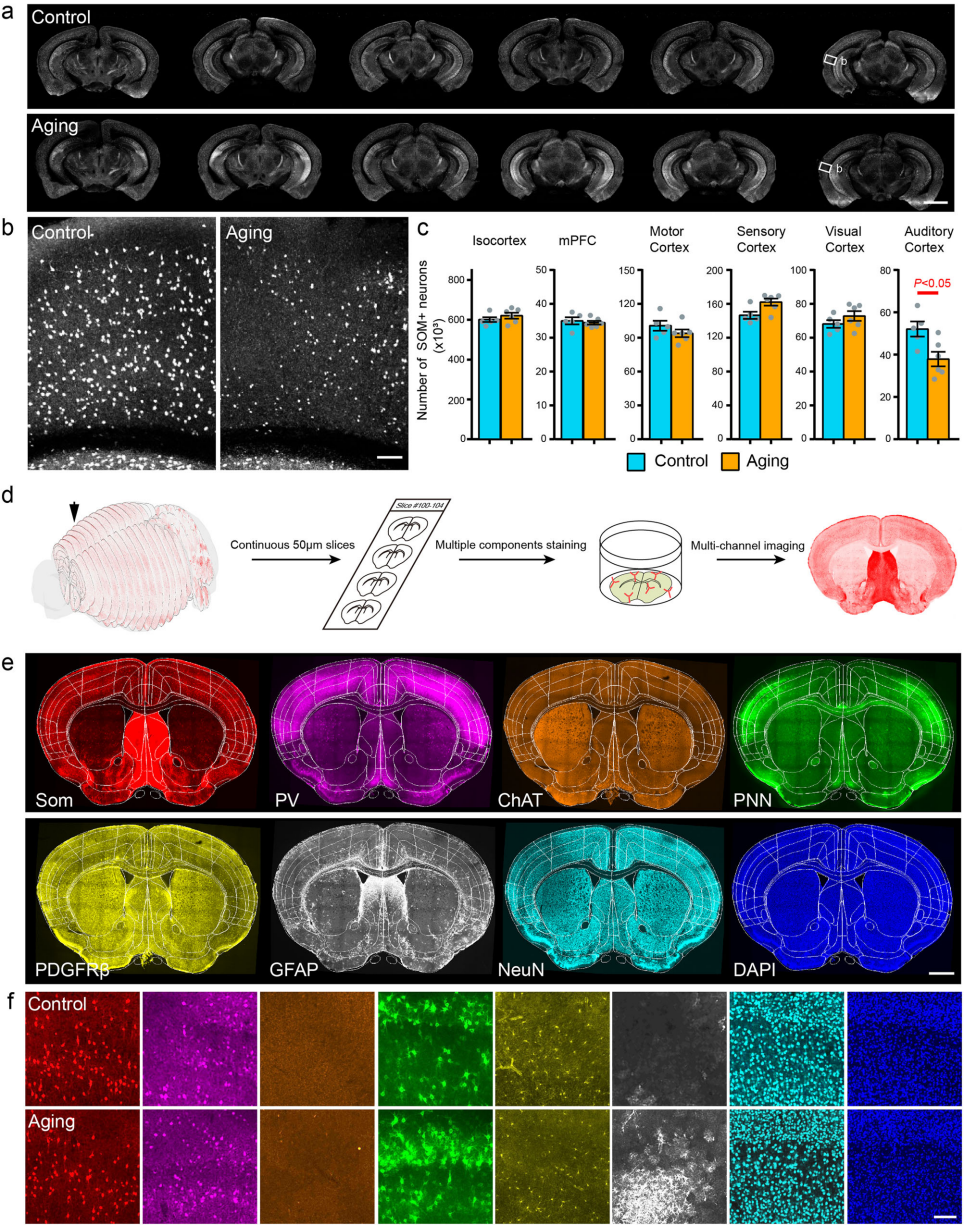
Region-specific changes of SOM neurons and its microenvironment in the mouse brain during aging. **a** Comparison of the intensity of somatostatin neurons between aging and normal adult mice (100 µm thickness). **b** Magnified view of the boxed region in A. **c** The number of somatostatin neurons in the isocortex and each segmented region. mPFC, medial prefrontal cortex. For control panels, *n* = 5 per group; for 14 months aging panels, *n* = 6 per group (95% confidence level, *P < 0.05; two-tailed Student’s t test). Scatter points represent the somatostatin neurons of each animal. Error bars, mean ± SEM. **d** Brain slices to be post-processed were selected from the obtained serial 50 μm sections, and multicomponents surrounding neurons were stained by immunofluorescence. After performing multichannel imaging on these brain slices respectively, these images are registered to the same brain slice using feature points of the slice outline. **e** Images of different components from four continuous brain slices registered to the Allen Brain Atlas (50 µm thickness). **f** Comparison the expression of different proteins in the auditory cortex between aging and normal adult mice. Scale bars: a, 2 mm; b, 100 μm; e, 1 mm, f, 200 μm.

When facing with age-related morphological changes, it is necessary to find differences from multiple samples. Meanwhile, in the process of structural analysis, excessive processing of samples in the early stage may lead to the loss of pathological features. Therefore, multi-samples structural dissection with intact tissue morphology is crucial for dissecting the changes in neurons and their surrounding multi-components during aging. In order to verify the convenience and stability of array-fMOST in multisamples comparison, we quantitatively analyzed the morphology of somatostatin neurons and neuronal microenvironment changes in the brains of naturally aging mice. We used 3- and 14-month-old SOM-Cre::Ai14 mice, which expressing red fluorescent signal in somatostatin positive neurons. The 14-month-old mice correspond to about 45-50 years old in humans, which is the group in the middle-aged period of the aging study. SOM-positive neurons are known to be the major interneuron population innervating the dendrites of pyramidal cells. It may be involved in functions such as dendritic plasticity, synchronization of rhythmic activity, and formation of new spatial memories^28^. The number of SOM neurons in the auditory cortex decreased significantly beginning in midlife^29^. However, the reason for this neuron loss phenomenon remains unknown. To address this problem, we used array-fMOST to compare whole-brain SOM-positive neurons in 14-month-old naturally aging and 3-month-old adult control mice (Fig. 4a). We found that, the number of SOM-positive neurons in the auditory cortex of the 14-month-old aging mice was decreased (Fig. 4b), which is consistent with previous studies. In order to accurately and quantitatively analyze the number of SOM-positive neurons in different regions of the whole brain, we used self-developed 2D slice registration software in our previous study^30^, to perform 2D segmentation of brain region boundaries in these data. Combining soma recognition and counting, we found that the number of SOM-positive neurons in the auditory cortex of 14-month-old mice was significantly reduced (Fig. 4c).

To explore the mechanism of the reduction of SOM-positive neurons in the auditory cortex of 14-month-old mice, we evaluated the composition of the SOM-positive neuronal microenvironment within the mouse auditory cortex and its changes in aging mice. After fluorescence imaging with array-fMOST, whole sections can be collected to be used for subsequent validation experiments, such as protein molecular phenotyping and super-resolution imaging. Here, we used immunofluorescence to stain multiple components within the SOM-positive neuronal microenvironment in serial sections. we retrieved four consecutive slices with a thickness of 50 μm one by one immediately after the sample was cut during array-fMOST imaging. The collected brain slices could correspond to the imaging layers in the whole-brain datasets. Therefore, 2D slices could be registered into 3D data by collecting signals from the system. Then we did immunostaining for one or two proteins in each brain slice. Through four kinds of staining on four consecutive brain slices, the structural characteristics of the same brain region could be analyzed. The obtained images were registered to the Allen Brain Atlas with brain area accuracy. Thus, the distribution of various components was summarized (Fig. 4d). The registered brain slices can realize the analysis and comparison of various components in the brain area.

As shown in Fig. 4e below, the first is the other types of neurons surrounding SOM neurons, then the extraneural matrix network (Perineuronal nets, PNN) of SOM neurons; the relevant components of neurovascular coupling around SOM neurons. Lastly, the entire neurons and cells in the auditory cortex. Compared with the normal adult group, the auditory cortex in 14-month-old mice showed higher expression of glial fibrillary acidic protein (GFAP), while the expression of platelet-derived growth factor (PDGFRβ) was significantly diminished (Fig. 4f). The above results clearly reveal the spatial pattern of cell loss in the mouse cortex and the changes in brain spatial information during natural aging. It shows that array-fMOST can screen pathological differences from multi-samples at single-cell resolution. At the same time, it can also realize molecular phenotypes identification after imaging, including post-imaging counterstaining of individual sections and registration between these adjacent slices. Such integrated high-dimensional information may accelerate our understanding of biological systems at multiple levels.

### Whole-brain input pattern of the medial prefrontal cortex and its changes in Alzheimer’s disease and autism model mice

**Fig. 5:**
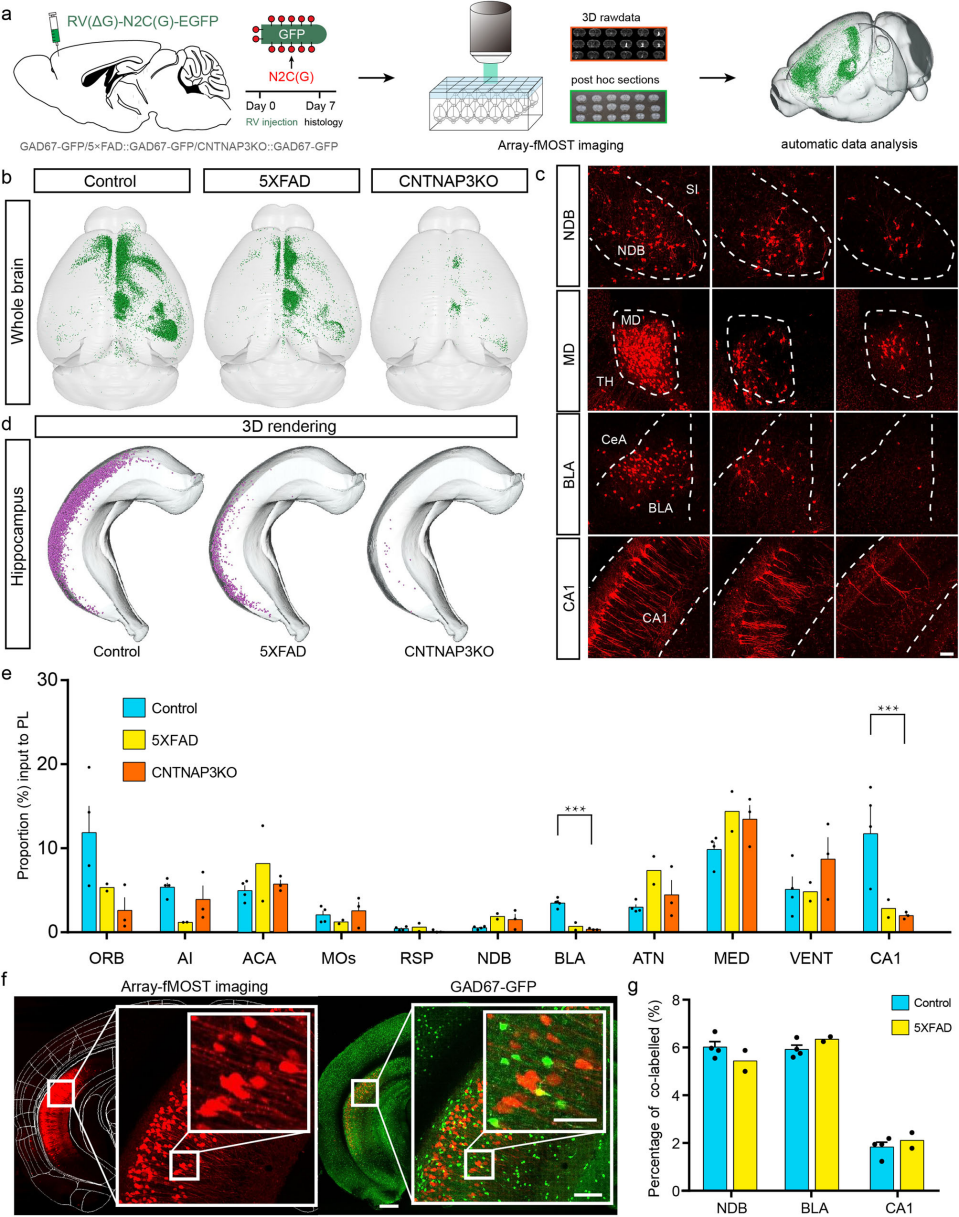
Whole brain accurate quantitative analysis of retrograde projection lesions of the medial prefrontal cortex neurons during AD and autism. **a** Diagram of the multi-samples labelling, array-fMOST imaging and data analyzing procedure under different pathological conditions. **b** 3D view of whole brain neurons projected to mPFC in control, 5×FAD and CNTNAP3KO mice. **c** Representative brain regions exhibiting decreased projection to the medial prefrontal cortex (mPFC) in 5×FAD and CNTNAP3KO mice are shown (100 µm thickness). **d** Reconstruction 3D view of brain region CA1 exhibiting decreased projection to mPFC in 5×FAD and CNTNAP3KO mice. **e** The proportion radio of neurons input to mPFC in these brain regions to the total number of neurons input to mPFC in the whole brain. For control panels, *n* = 4; 5×FAD panels, n = 2; CNTNAP3KO panels, *n* = 3. (95% confidence level **P < 0.01, ***P < 0.001, based on one-way repeated-measures (RM) ANOVA with Tukey’s post hoc test). Scatter points represent the proportion input to mPFC of each animal. Error bars, mean ± SEM. **f** Dual-channel imaging revealing neurons input to mPFC areas were GAD67 negative in CA1. **g** Percentage of RV and GAD67 co-labelled neurons to RV-labelled neurons in these brain regions. For control panels, *n* = 4; 5×FAD panels, *n* = 2. Error bars, mean ± SEM. Scale bars: **c**, 100 μm; **f**, 500 μm, 100 μm, 50 μm.

To evaluate the scalability and applicability of array-fMOST in three-dimensional cellular spatial localization analysis, we measured the whole-brain input pattern of the medial prefrontal cortex (mPFC) and its lesions in Alzheimer’s disease and autism model mice. First, we acquired the input pattern of mPFC in normal adult mice. Then, we used 5×FAD and CNTNAP3KO mice to model the manifestations of Alzheimer’s disease and autism. Heterozygous male 5×FAD mice and CNTNAP3KO mice were crossed with female GAD67-GFP mice to detect the co-labelled ratio and analyze the neuron types of lesion circuits. Next, we injected 200 nl RV-N2C(G)-ΔG-mCherry in the mPFC of GAD67-GFP, 5×FAD::GAD67-GFP and CNTNAP3KO::GAD67-GFP mice, respectively to label their whole-brain input neurons. Subsequently, we acquired the spatial location of input neurons in the whole brain via dual-channel imaging (Fig. 5a).

Compared with control group, the number of input neurons in several brain regions of 5×FAD and CNTNAP3KO mice decreased considerably, including the hippocampal region, and amygdaloid nuclei (Fig. 5b & c). This indicated that the two circuits from the CA1 and BLA to mPFC may be damaged in Alzheimer’s disease and autism model mice. In order to realize the spatial localization and identification of lesion circuits, we extracted the spatial information of input neurons in the hippocampal region and reconstructed in the 3D view. As shown in Fig. 5d, we observed a reduction of cell density throughout the lateral hippocampus in 5×FAD and CNTNAP3KO mice. Next, to identify the molecular phenotype of neurons within CA1 that project to the mPFC. We merged mCherry-labeled neurons with GFP-labeled GAD67 neurons using the GAD67-GFP channel in dual-color imaging. From the results we found that the majority of mCherry-labeled neurons were GAD67 negative (Fig. 5f). By calculating the co-labelled ratio, we found that less than 10% of the neurons projected to mPFC from these brain regions, such as NDB, BLA, CA1, were GAD67 positive neurons (Fig. 5g). This suggests that the majority neurons in these three regions projecting to the mPFC were excitatory. In 5×FAD and CNTNAP3KO mice, these CA1 excitatory connections to the mPFC were significantly reduced (Fig. 5e). Both of these results clearly reveal a spatial pattern of cell connections decrease in the 3D space of the whole brain as well as the hippocampus in Alzheimer’s disease and autism model mice. These results indicate that this imaging and data analysis pipeline can be readily applied to other mouse models of disease, offering a highly versatile platform to study in situ mechanisms of disease initiation and progression

## Discussion

Anatomical dissection of multiple intact tissues is expected to provide valuable insights into the mechanisms underlying neurobiological function and dysfunction. In order to achieve the anatomical information at single-cell resolution from multiple samples simultaneously, we developed a high-throughput cell localization and identification system, which contains array embedding, large-scale imaging, post-imaging staining and data analysis. This approach is conducive to the comparison of differences between multiple samples under different pathological conditions.

During sample preparation, agarose was used to embed the multiple samples such as mouse brains. Based on embedding for single mouse brain in our previous study^31^, we use the freezing point of molten agarose to array multisamples in specific space, which facilitates subsequent parallel imaging. It does not require additional tissue processing, such as dehydration, degreasing, and refractive index matching. Then, it avoids the tissue shrinkage and expansion, especially for the nonuniform in deep structures of the whole organ^20^. The anatomical structure and biological macromolecular of the samples remain intact during agarose-embedding^30^, which can be identified and quantitatively analyzed for different pathologic or physiological conditions.

The most attractive point of this method is the ability to image on multiple samples simultaneously, which increases the efficiency when comparing different samples. To quickly analyze the results, we set the resolution of the current imaging system at 0.65 μm × 0.65 μm × 3 μm. Meanwhile, the imaging process is continuous in situ, which guarantees the integrity of sample information and self-registration characteristics. Therefore, the three-dimensional data obtained by this method could be used for axon tracing.

With post-treatments, array-fMOST can provide a reliable way to obtain integrated high-dimensional information from intact biological samples. After acquiring 3D data, the serial tissue sections can be collected for immunohistochemical staining and other processes. Determine the location of interest by referring to the whole-brain imaging data, extract the corresponding section for staining of various proteins. Then, these images obtained by serial section staining are registered to the same brain slice, which provides a new tool for analyzing the structural relationship and changing of various components in three-dimensional brain space. Unlike methods such as SWITCH^32^, which perform multiple rounds of staining and elution on a single tissue, we use mutual registration between consecutive slices to achieve near-in situ analysis of multiplex information. We focused more on the localization of cellular bodies and the distribution of proteins in the brain region than on the subcellular level of multiprotein colocalization. In the future, we hope to be able to achieve precise subcellular identification, which would thereby allow precise mapping of the expression of biomarkers of interest in specific cell types at the subcellular level, although it would be challenging. Using the registered dataset, we successfully performed unbiased combinatorial expression analysis of neuropeptide somatostatin-positive neurons and neuronal microenvironments in aging and normal adult mice to unequivocally identify diverse component alterations based on their distinct protein expression patterns.

In the study, we optimize the structure and parameters of our self-made vibrating slicer to reduce the parasitic movement of the 57mm-length blade to several micrometers when the slicer is working^13^. So, we can obtain a precise section of the whole agarose-embedding multi-samples array with about 12 microns thick. Combining the HD-fMOST optical section method with the precise section, we realize cell-resolution 3D imaging without the degradation caused by tissue absorption and scattering. Compared with imaging the whole mouse brain one by one, the strategy for parallel imaging greatly reduce the time spent for imaging scanning, tissue section, and imaging system adjustment. We process the big data produced by array-fMOST through a high-performance computer cluster and parallelization algorithms. Some key parameters like image redundancy, intensity correction parameters and so on, which are used for data process can be reused for all the samples because the samples are imaged on the same fMOST system at the same time. So, the average processing time for each sample is also significantly reduced for array-fMOST.

The array-fMOST has the potential to be combined with the embedding scheme of tissue clearing and PNAGA to deepen the imaging monolayer scanning depth and slice thickness^33^. On the premise of stably acquiring continuous 3D data, this will improve the acquiring efficiency by reducing the time spent on imaging and cutting.

This pipeline can also be applied to complete 3D continuous dataset acquisition of the brain and spinal cord. Visualizing cellular distribution and circuit connectivity in intact brain and spinal cord structures up to 7 cm in length remains challenging with existing techniques. Traditional histological methods are prohibitively intensive manual labor; Manual section collection, staining, and imaging of hundreds or even thousands of sections while maintaining sample order and location is labor-intensive and costly^34^. Whereas our protocol could provide a way to embed contiguous tissue from the entire brain and spinal cord in parallel within a single agarose block, allowing acquisition and sectioning of the intact tissue.

Together with its simplicity, scalability, and broad applicability, our data suggests that array-fMOST provides new opportunities for combined multi-sample whole-organ imaging and molecular phenotypes identification analysis together, to study pathogenesis and progression of disease in situ.

## Methods

### Animals

Thy1-GFP M-line mice (#007788, Jackson Laboratory, Bar Harbor, ME, USA), Thy1-YFP H-line mice (#003782, Jackson Laboratory) and GAD67-GFP mice (#007677, Jackson Laboratory) were from Jackson Laboratory. Twelve 2-month-old male Thy1-GFP M-line mice and Twelve 2-month-old male Thy1- YFP H-line mice were used in Fig. 1d and Fig. 3a. The SOM::tdTomato mouse line was generated by crossing SOM-IRES-Cre mice, in which Cre expression is driven by the endogenous SOM promoter/enhancer elements (#013044, Jackson Laboratory), with Cre-dependent tdTomato mice (Ai14, #007908, Jackson Laboratory). A total of twelve mice of 2-, and 14-month-old female SOM-Cre::Ai14 mice, six 14-month-old female 5×FAD::SOM-Cre::Ai14 mice were used in Fig. 3b and Fig. 4.

The 5×FAD mouse line expresses the human APP and PSEN1 transgenes^35,36^. CNTNAP3KO mouse was a gift from Zilong Qiu Lab^37^. Heterozygous male 5×FAD mice and CNTNAP3KO mice were crossed with female GAD67-GFP mice. Four 12-month-old male GAD67-GFP mice, two 12-month-old male 5×FAD:: GAD67-GFP mice and three 12-month-old male CNTNAP3KO:: GAD67-GFP mice were used in Fig. 5.

All mice used in this study were housed in normal cages in an environment with a 12-h light/dark cycle with food and water *ad libitum*. All animal experiments were approved by the Animal Ethics Committee of the Huazhong University of Science and Technology.

### Fluorescein-labeled tomato (Lycopersicon Esculentum) lectin staining

Fluorescein-labeled tomato lectin (FL-1171, Vector Laboratories, Burlingame, CA, USA) is an effective marker of blood vessels. To visualize the whole-brain vascular structures, endothelial cells were labelled by tail intravenous injection of fluorescein-labelled tomato lectin according to the method described previously^38^.

### Virus injection

RV-N2C(G)-ΔG-mCherry (3.00E + 08 IFU/mL) were purchased from Wuhan BrainVTA Co., Ltd., China. All mice were deeply anesthetized by intraperitoneally injected (100 g/ml) with 2% chloral hydrate and 10% urethane-configured anesthetic before they were mounted and microinjected with a stereotaxic system. For retrograde monosynaptic tracing, 150 nl of RV-N2C(G)-ΔG- mCherry was injected into the PL (bregma 1.9 mm, lateral 0.3 mm, depth 2.3 mm from skull surface) of GAD67-GFP, 5×FAD::GAD67-GFP and CNTNAP3KO::GAD67-GFP mice. After the surgery, the incisions were stitched and lincomycin hydrochloride and lidocaine hydrochloride gel was applied to prevent inflammation and alleviate pain for the animals. The mice injected with virus were killed for analysis 1 week after the injection.

### Tissue preparation

All histological procedures were conducted as follows: the mice were anaesthetised by intraperitoneal injection with 1% sodium pentobarbital solution, and subsequently intracardially perfused with 0.01 M phosphate-buffered saline (PBS, Sigma-Aldrich, USA, P3813), then followed by 4% paraformaldehyde (PFA, Sigma-Aldrich, USA, 158127) in 0.01 M PBS. The brains were then excised and post-fixed in 4% PFA at 4 °C for 24 h. After fixation, each intact brain was rinsed overnight at 4 °C in 0.01 M PBS.

### Agarose embedding

The prepared mouse brain was embedded in agarose^31^. The brains were dried and embedded in melted oxidised agarose using a silicone mould. For multiple samples, we set samples in an array arrangement. As an example, after rinsing with PBS, twenty-four fixed mouse brain samples were divided into four groups according to different pathological or labelling methods, with six mouse brains in each group. The first group of six mouse brain samples were placed in the mould, ventral side down. There were several cuboid-shaped grooves in the mould for brain embedding and gridlines for correcting the brain orientation. After adjusting the orientation of the mouse brain, add molten liquid agarose to the mould to cover the surface of the mouse brain. After waiting for about three minutes, when the surface of the agarose in the mould begins to solidify, and the agarose around the sample is still in a liquid state. At this time, a second group of six mouse brains is placed on the surface of the agarose (Fig. 1b). Repeat this step several times until all mouse brains are placed in the mold. The mould and brains were placed in a 55 °C water bath for 0.5 hour until the surfaces of the brains were fully coated with agarose. During the water bath, the orientation of the brain could be easily adjusted to ensure the correct sectioning angle of the whole brain. Then, the mould and brains were left at room temperature for 0.5 hours to allow the agarose to solidify.

### array-fMOST whole-brain imaging

A schematic diagram of our imaging and section system is shown in Fig. 1c. Two lasers (488 nm and 561 nm, 06-MLD, Cobolt, Sweden) were combined by a dichroic mirror (DM1: ZT502rdc, Chroma, USA). The combined laser beams were then expanded to about 25 mm wide by a pair of lenses (L1: AC050-008-A, Thorlabs, USA; L2: AC254-250-A, Thorlabs, USA). We used a cylindrical lens (C1: ACY254-100-A, Thorlabs, USA) to focus the expended laser beams in one direction. Then the linear beams were projected to the focus plane of the objective by the combination of an achromat lens (L3: AC254-125-A, Thorlabs, USA), an excitation filter (F1: ZET405/488/561x, Chroma, USA), a dichroic mirror (DM2: ZT405/488/561rpc, Chroma, USA) and a 10× objective (OBJ: NA 0.6, XLPLN10XSVMP, Olympus, Japan). After filtered by the dichroic mirror and an emission filter (F2; ZET405/488/561 m, Chroma, USA), the emission signals collected by the objective were focused onto the detective plane of the scientific complementary metal-oxide-semiconductor transistors (sCMOS: C13440-20CU, Hamamatsu, Japan) by a tube lens (TL, U-TLU, Olympus, Japan). A fluorescence filter cube (DM3: ZT561rdc, Chroma, USA; F3: ET590lp, Chroma, USA; F4: AT525/30 m, Chroma, USA) split the detected signals into two channels with different wavelengths. We operated the sCMOS in the sub-array mode as a multi-line detector to remove the background^12^. Volume imaging was achieved by using a high-accuracy 3D stage for imaging scanning and a vibrating unit for section with a 57 mm wide blade.

### Immunohistochemistry and imaging

As for immunostaining, we retrieved four consecutive slices one by one immediately after the sample was cut during array-fMOST imaging. Collected brain slices could correspond to the imaging layers in the whole-brain datasets. Therefore, 2D slices could be registered into 3D data by collecting signals from the system. Then the four consecutive sections were subjected to the conventional immunohistochemical process^30^. We did immunostaining for one or two proteins in each brain slice. Through four kinds of staining on four consecutive brain slices, the structural characteristics of the same brain region could be analysed.

The prepared slices were washed using PBS for 3 × 10 min, permeabilised using PBST (0.3% Triton-X-100 in PBS) for 1 h, and then incubated with blocking solution (5% bovine serum albumin (BSA) in PBST) for 1 h followed by incubation with primary antibodies overnight at 4 °C. After washing the slices 3 × 10 min in PBS, the appropriate secondary antibodies were applied for 2 h at room temperature.

Immunofluorescence staining of two proteins was performed on the first section. Antibodies included mouse anti-PV (Millipore, MAB1572, 1:1000 dilution), and Wisteria Floribunda Lectin (Vectorlabs, FL-1351-2, 1:200 dilution). Corresponding to this, the secondary antibody used was Alexa Fluor 647 donkey anti-mouse IgG (Invitrogen, A31571, 1:1000 dilution). As for the second slice, antibodies included goat anti-ChAT (Millipore, AB144P, 1:800 dilution), mouse anti-NeuN (Covance, SIG-39860, 1:1000 dilution) and Alexa Fluor 488 donkey anti-goat IgG (Invitrogen, A11055, 1:1000 dilution), Alexa Fluor 647 donkey anti-mouse IgG (Invitrogen, A31571, 1:1000 dilution). As for the third slice, antibodies included rabbit anti-GFAP (Abcam, ab7260, 1:1000 dilution) and Alexa Fluor 647 donkey anti-rabbit IgG (Invitrogen, A31573, 1:1000 dilution). As for the fourth slice, antibodies included rabbit anti-PDGFRβ (Abcam, ab32570, 1:800 dilution) and Alexa Fluor 647 donkey anti-rabbit IgG (Invitrogen, A31573, 1:1000 dilution). All sections collected were subsequently stained with DAPI (Thermofisher, D1306, 0.5 μg/mL). After immunostaining, we used anti-fluorescence attenuation mounting tablets to mount the slides the sections. Finally, slices were imaged with a Leica SP8 confocal microscope (20×, 0.75 NA) and processed using ImageJ software (National Institutes of Health, Bethesda, MD, United States).

### Data processing and registration

The stripe-shaped raw data were firstly stitched together into entire coronal section images. Then the shading was removed by illumination correction. The grey value of pixels in each row were accumulated as a signal series. The series was smoothed twice independently with different spans, and the ratios of smoothed series were used as illumination correction coefficient. For whole-brain consecutive imaging, to quantify and integrate the whole-brain connections, the coordinates of the soma of input neurons were registered to Allen CCFv3 using the transformation parameters acquired by the previously described methods^39^. Briefly, we segmented several brain regions as landmarks through cytoarchitecture references, such as the outline, caudoputamen, medial habenula, lateral ventricle, and third ventricle. Based on these landmarks, we performed affine transformation and symmetric image normalization in Advanced Normalization Tools (ANTS) to acquire transformation parameters. Using the self-developed analysis software described in our previous article^30^, we registered four consecutive slices after staining to the Allen Mouse Brain Common Coordinate Framework, which is compatible with multimodal images and slant section planes.

### Quantitative analysis

We automatically identified and localized the soma of input neurons using NeuroGPS^40^ and manually checked the results to eliminate indiscernible mistakes. Next, we warped the soma coordinates to Allen CCFv3^41^ using the transformation parameters from the aforementioned registration. Finally, we calculated the number and proportion of input neurons in each brain region of interest to generate the quantified whole-brain inputs. We visualized the data set using Amira software (v.5.2.2, FEI) to generate the figures. The data set acquired by the dual-colour precise imaging system was separated into the GFP channel and RFP channel. The registered data was imported into Amira to generate the outline of the mouse brain.

For two-dimensional slice cell recognition, the images were sequentially filtered with top-hat operation, binarization, and opening operations. The resolution of the images used in this study was 0.65 μm/pixel, therefore, we chose a disk-shaped structure element with a radius of 9 pixels for the top-hat filter and 5 pixels for the opening filter. For binarization, the threshold depends on the signal-to-background ratio and the absolute intensity of the image, and we selected 30 as the fluorescence intensity threshold. When all slices were recognised and registered, the number of labelled neurons in each region was counted automatically. This process has been described before^30^.

### Statistics and reproducibility

To analyse the morphology of somatostatin neurons and neuronal microenvironment changes in the brains of naturally aging mice, a total of twelve mice of 2-, and 14-month-old female SOM-Cre::Ai14 mice were used. To measure the whole-brain input pattern of the medial prefrontal cortex (mPFC) and its lesions in Alzheimer’s disease and autism model mice, we used four 12-month-old male GAD67-GFP mice, two 12-month-old male 5×FAD:: GAD67-GFP mice and three 12-month-old male CNTNAP3KO:: GAD67-GFP mice.

All graphs were generated using Prism v8.0 (GraphPad, La Jolla, CA, USA), and the data were analysed with the two-tailed t-test using Prism. The confidence level (P value) was set to 0.05, and the results are presented as mean ± standard error of the mean.

### Reporting summary

Further information on research design is available in the Nature Portfolio Reporting Summary linked to this article.

## Supplementary information

Supplementary Information

Description of Additional Supplementary Files

Supplementary Data 1

Reporting Summary

## Data Availability

The source data for Figs. 4c, 5e ang 5 g are provided in Supplementary Data 1. All other data are available at our website for free download (http://atlas.brainsmatics.org/a/chen2212).
