# Supplementary material for: Three-dimensional mapping in multi-samples with large-scale imaging and multiplexed post staining
Source: Commun Biol. 2023 Feb 3;6:148. doi: 10.1038/s42003-023-04456-3 (PMC9898531; doi:10.1038/s42003-023-04456-3)
Supplement: Supplementary file 1 — Supplementary Information [file 42003_2023_4456_MOESM1_ESM.pdf]

Supplementary materials

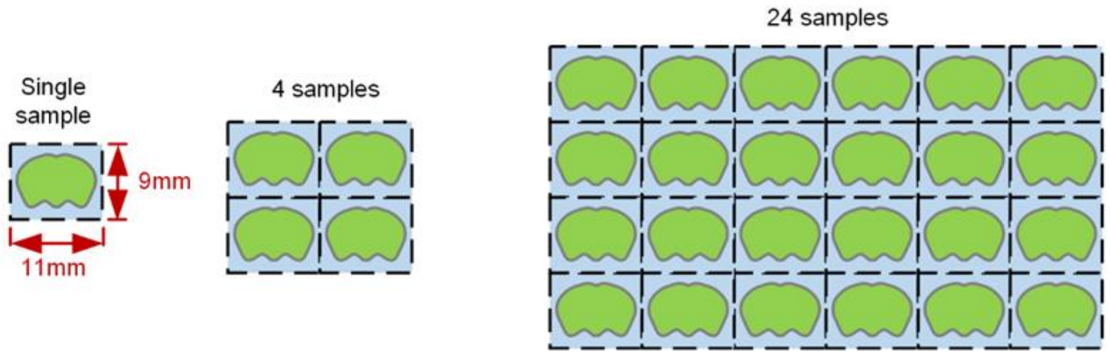

Supplementary Figure 1. The arrangement of different quantities of samples

	Single sample	4 samples	24 samples
Height of samples	12 mm		
Interval of imaging	3 μm		
Number of imaging planes	4000		
Imaging time per plane	12.9 s	40.3 s	210.8 s
Total imaging time	14.4 h	44.8 h	234.2 h
Interval of slicing	12 μm		
Number of slices	1000		
Vibrating section time per slice	35 s	57 s	145 s
Total slicing time	9.7 h	15.8 h	40.3 h
Total time expenditure	24.1 h	60.6 h	274.5 h
Average time expenditure per sample	24.1 h	15.2 h	11.4 h

Supplementary Table 1. Comparison of time expenditure of different quantities of samples using array-fMOST 1.
