# Supplementary material for: Three-dimensional mapping in multi-samples with large-scale imaging and multiplexed post staining
Source: Commun Biol. 2023 Feb 3;6:148. doi: 10.1038/s42003-023-04456-3 (PMC9898531; doi:10.1038/s42003-023-04456-3)
Supplement: Supplementary file 2 — Description of Additional Supplementary Files [file 42003_2023_4456_MOESM2_ESM.pdf]

Description of Additional Supplementary Files

File name: Supplementary Data 1

Description: The source data behind the Figure 4c, 5e ang 5g in the paper
