# Supplementary material for: Three-dimensional mapping in multi-samples with large-scale imaging and multiplexed post staining
Source: Commun Biol. 2023 Feb 3;6:148. doi: 10.1038/s42003-023-04456-3 (PMC9898531; doi:10.1038/s42003-023-04456-3)
Supplement: Supplementary file 4 — Reporting Summary [file 42003_2023_4456_MOESM4_ESM.pdf]

nature portfolio

Corresponding author(s): Xiangning Li

Last updated by author(s): Nov 25, 2022

# Reporting Summary

Nature Portfolio wishes to improve the reproducibility of the work that we publish. This form provides structure for consistency and transparency in reporting. For further information on Nature Portfolio policies, see our Editorial Policies and the Editorial Policy Checklist.

## Statistics

For all statistical analyses, confirm that the following items are present in the figure legend, table legend, main text, or Methods section.

| n/a | Confirmed | |
|---|---|---|
| ☐ | ☒ | The exact sample size (*n*) for each experimental group/condition, given as a discrete number and unit of measurement |
| ☐ | ☒ | A statement on whether measurements were taken from distinct samples or whether the same sample was measured repeatedly |
| ☐ | ☒ | The statistical test(s) used AND whether they are one- or two-sided<br>*Only common tests should be described solely by name; describe more complex techniques in the Methods section.* |
| ☒ | ☐ | A description of all covariates tested |
| ☐ | ☒ | A description of any assumptions or corrections, such as tests of normality and adjustment for multiple comparisons |
| ☐ | ☒ | A full description of the statistical parameters including central tendency (e.g. means) or other basic estimates (e.g. regression coefficient) AND variation (e.g. standard deviation) or associated estimates of uncertainty (e.g. confidence intervals) |
| ☐ | ☒ | For null hypothesis testing, the test statistic (e.g. *F*, *t*, *r*) with confidence intervals, effect sizes, degrees of freedom and *P* value noted<br>*Give P values as exact values whenever suitable.* |
| ☒ | ☐ | For Bayesian analysis, information on the choice of priors and Markov chain Monte Carlo settings |
| ☒ | ☐ | For hierarchical and complex designs, identification of the appropriate level for tests and full reporting of outcomes |
| ☒ | ☐ | Estimates of effect sizes (e.g. Cohen's *d*, Pearson's *r*), indicating how they were calculated |

*Our web collection on statistics for biologists contains articles on many of the points above.*

## Software and code

Policy information about availability of computer code

| | |
|---|---|
| Data collection | The software and codes used for imaging, imaging preprocessing, visualization and registration in this study were similar to previous studies (Sun et al, 2022, Nature communications, doi: 10.1038/s41467-022-28493-4; Zhong et al, 2022, Nature Methods, doi:10.1038/s41592-021-01074-x; Zhou et al, 2021, Science Bulletin, https://doi.org/10.1016/j.scib.2021.08.003 ) and details were described in Methods. Cell counting was performed by NeuroGPS (Quan et al, 2013, Scientific Reports, 10.1038/srep01414) and code in previous study (Chen et al, 2021, Frontiers in Neuroanatomy, doi: 10.3389/fnana.2021.771229). Amira software (v 5.2.2, FEI, France) was used for data visualization. Confocal images were collected via LAS_X software of Leica. |
| Data analysis | MatLab (R2021b) was used for two-dimensional slice cell recognition and registration. Statistical analysis was performed using preset algorithms in Graphpad Prism (v.6). Figures were prepared in Adobe Illustrator CC2017. |

For manuscripts utilizing custom algorithms or software that are central to the research but not yet described in published literature, software must be made available to editors and reviewers. We strongly encourage code deposition in a community repository (e.g. GitHub). See the Nature Portfolio guidelines for submitting code & software for further information.

## Data

Policy information about availability of data

All manuscripts must include a data availability statement. This statement should provide the following information, where applicable:

- Accession codes, unique identifiers, or web links for publicly available datasets
- A description of any restrictions on data availability
- For clinical datasets or third party data, please ensure that the statement adheres to our policy

All the data that support the findings of this study are provided in the article and its Supplementary information files. Source data are provided with this paper. Additional information about this paper are available from the corresponding author upon reasonable request.

## Human research participants

Policy information about studies involving human research participants and Sex and Gender in Research.

| | |
|---|---|
| Reporting on sex and gender | *Use the terms sex (biological attribute) and gender (shaped by social and cultural circumstances) carefully in order to avoid confusing both terms. Indicate if findings apply to only one sex or gender; describe whether sex and gender were considered in study design whether sex and/or gender was determined based on self-reporting or assigned and methods used. Provide in the source data disaggregated sex and gender data where this information has been collected, and consent has been obtained for sharing of individual-level data; provide overall numbers in this Reporting Summary. Please state if this information has not been collected. Report sex- and gender-based analyses where performed, justify reasons for lack of sex- and gender-based analysis.* |
| Population characteristics | *Describe the covariate-relevant population characteristics of the human research participants (e.g. age, genotypic information, past and current diagnosis and treatment categories). If you filled out the behavioural & social sciences study design questions and have nothing to add here, write "See above."* |
| Recruitment | *Describe how participants were recruited. Outline any potential self-selection bias or other biases that may be present and how these are likely to impact results.* |
| Ethics oversight | *Identify the organization(s) that approved the study protocol.* |

Note that full information on the approval of the study protocol must also be provided in the manuscript.

# Field-specific reporting

Please select the one below that is the best fit for your research. If you are not sure, read the appropriate sections before making your selection.

☒ Life sciences ☐ Behavioural & social sciences ☐ Ecological, evolutionary & environmental sciences

For a reference copy of the document with all sections, see nature.com/documents/nr-reporting-summary-flat.pdf

# Life sciences study design

All studies must disclose on these points even when the disclosure is negative.

| | |
|---|---|
| Sample size | No statistical methods were used to predetermine sample size; |
| Data exclusions | No data were excluded. |
| Replication | Results described throughout the paper were reproduced. 5-10 rounds of experimentation were performed in independent animals. The n value in the paper indicated the times of replication. No results were included that were not observed in multiple animals. No issues were identified in replicating any of the reported findings |
| Randomization | For all experiments in the present study, the animals were randomly assigned to experimental group and control group. For the comparison between AD group, autism group and wild type group, the hemizygous 5XFAD mice and CNTNAP3KO mice were randomly assigned to the AD group and autism group. The noncarriers of AD mutant genes from same genetic background were randomly assigned to the wild type group. |
| Blinding | For the comparison of neuron number between aging mice and control group, the investigator was blinded to the groups. For rabies tracing in analyzing whole-brain input pattern of the medial prefrontal cortex (mPFC) and its lesions, the person who analyzed the data was binded to the strain of the animals. |

# Reporting for specific materials, systems and methods

We require information from authors about some types of materials, experimental systems and methods used in many studies. Here, indicate whether each material, system or method listed is relevant to your study. If you are not sure if a list item applies to your research, read the appropriate section before selecting a response.

## Materials & experimental systems

| n/a | Involved in the study |
|---|---|
| ☐ | ☒ Antibodies |
| ☒ | ☐ Eukaryotic cell lines |
| ☒ | ☐ Palaeontology and archaeology |
| ☐ | ☒ Animals and other organisms |
| ☒ | ☐ Clinical data |
| ☒ | ☐ Dual use research of concern |

## Methods

| n/a | Involved in the study |
|---|---|
| ☒ | ☐ ChIP-seq |
| ☒ | ☐ Flow cytometry |
| ☒ | ☐ MRI-based neuroimaging |

## Antibodies

Antibodies used

Immunofluorescence staining of two proteins was performed on the first section. Antibodies included mouse anti-PV (Millipore, MAB1572, 1:1000 dilution), and Wisteria Floribunda Lectin (Vectorlabs, FL-1351-2, 1:200 dilution). Corresponding to this, the secondary antibody used was Alexa Fluor 647 donkey anti-mouse IgG (Invitrogen, A31571, 1:1000 dilution). As for the second slice, antibodies included goat anti-ChAT (Millipore, AB144P, 1:800 dilution), mouse anti-NeuN (Covance, SIG-39860, 1:1000 dilution) and Alexa Fluor 488 donkey anti-goat IgG (Invitrogen, A11055, 1:1000 dilution), Alexa Fluor 647 donkey anti-mouse IgG (Invitrogen, A31571, 1:1000 dilution). As for the third slice, antibodies included rabbit anti-GFAP (Abcam, ab7260, 1:1000 dilution) and Alexa Fluor 647 donkey anti-rabbit IgG (Invitrogen, A31573, 1:1000 dilution). As for the fourth slice, antibodies included rabbit anti-PDGFRβ (Abcam, ab32570, 1:800 dilution) and Alexa Fluor 647 donkey anti-rabbit IgG (Invitrogen, A31573, 1:1000 dilution). All sections collected were subsequently stained with DAPI (Thermofisher, D1306, 0.5 μg/mL). After immunostaining, we used anti-fluorescence attenuation mounting tablets to mount the slides the sections. Finally, slices were imaged with a Leica SP8 confocal microscope (20×, 0.75 NA) and processed using ImageJ software (National Institutes of Health, Bethesda, MD, United States).

Validation

There are all well characterized commercial antibodies. The specificity of the primary and secondary antibodies was validated by the manufactures.
PV:https://www.merckmillipore.com/CN/zh/product/Anti-Parvalbumin-Antibody,MM_NF-MAB1572?ReferrerURL=https%3A%2F%2Fcn.bing.com%2F&bd=1
Wisteria Floribunda Lectin: https://www.2bscientific.com/Products/VECT/FL-1351-2/Wisteria-Floribunda-Lectin-WFA-WFL-Fluorescein
ChAT: https://www.merckmillipore.com/CN/zh/product/Anti-Choline-Acetyltransferase-Antibody,MM_NF-AB144P?ReferrerURL=https%3A%2F%2Fcn.bing.com%2F&bd=1
NeuN: https://www.biolegend.com/ja-jp/products/purified-anti-fox3-neun-antibody-10823?GroupID=BLG15643
GFAP: https://www.abcam.cn/gfap-antibody-ab7260.html
PDGFRβ: https://www.abcam.cn/pdgfr-alpha--pdgfr-beta-antibody-y92-c-terminal-ab32570.html
Alexa Fluor 647 donkey anti-mouse IgG: https://www.thermofisher.cn/cn/zh/antibody/product/Donkey-anti-Mouse-IgG-H-L-Highly-Cross-Adsorbed-Secondary-Antibody-Polyclonal/A-31571
Alexa Fluor 488 donkey anti-goat IgG: https://www.thermofisher.cn/cn/zh/antibody/product/Donkey-anti-Goat-IgG-H-L-Cross-Adsorbed-Secondary-Antibody-Polyclonal/A-11055
Alexa Fluor 647 donkey anti-rabbit IgG:https://www.thermofisher.cn/cn/zh/antibody/product/Donkey-anti-Rabbit-IgG-H-L-Highly-Cross-Adsorbed-Secondary-Antibody-Polyclonal/A-31573

## Animals and other research organisms

Policy information about studies involving animals; ARRIVE guidelines recommended for reporting animal research, and Sex and Gender in Research

Laboratory animals

Thy1-GFP M-line mice (#007788, Jackson Laboratory, Bar Harbor, ME, USA), Thy1-YFP H-line mice (#003782, Jackson Laboratory) and GAD67-GFP mice (#007677, Jackson Laboratory) were from Jackson Laboratory. Twelve 2-month-old male Thy1-GFP M-line mice and Twelve 2-month-old male Thy1- YFP H-line mice were used in Fig.1 D and Fig.3 A. The SOM::tdTomato mouse line was generated by crossing SOM-IRES-Cre mice, in which Cre expression is driven by the endogenous SOM promoter/enhancer elements (#013044, Jackson Laboratory), with Cre-dependent tdTomato mice (Ai14, #007908, Jackson Laboratory). A total of twelve mice of 2-, and 14-month-old female SOM-Cre::Ai14 mice, six 14-month-old female 5×FAD::SOM-Cre::Ai14 mice were used in Fig.3 B and Fig.4.
The 5×FAD mouse line expresses the human APP and PSEN1 transgenes35, 36. CNTNAP3KO mouse was a gift from Zilong Qiu Lab37. Heterozygous male 5×FAD mice and CNTNAP3KO mice were crossed with female GAD67-GFP mice. Four 12-month-old male GAD67-GFP mice, two 12-month-old male 5×FAD:: GAD67-GFP mice and three 12-month-old male CNTNAP3KO:: GAD67-GFP mice were used in Fig. 5.
All mice used in this study were housed in normal cages in an environment with a 12-h light/dark cycle with food and water ad libitum. All animal experiments were approved by the Animal Ethics Committee of the Huazhong University of Science and Technology.

| | |
|---|---|
| Wild animals | The study did not involve wild animals |
| Reporting on sex | Female and male mice were used in this study |
| Field-collected samples | The study did not involve data collected from the field |
| Ethics oversight | All animal experiments were approved by the Animal Ethics Committee of the Huazhong University of Science and Technology. |

Note that full information on the approval of the study protocol must also be provided in the manuscript.
